# Indoor Localization Methods for Smartphones with Multi-Source Sensors Fusion: Tasks, Challenges, Strategies, and Perspectives

**DOI:** 10.3390/s25061806

**Published:** 2025-03-14

**Authors:** Jianhua Liu, Zhijie Yang, Sisi Zlatanova, Songnian Li, Bing Yu

**Affiliations:** 1 Mobile Geospatial Big Data Cloud Service Innovation Team, School of Geomatics and Urban Spatial Information, Beijing University of Civil Engineering and Architecture, Beijing 102616, China; 2108160322006@stu.bucea.edu.cn (Z.Y.); 2108570023119@stu.bucea.edu.cn (B.Y.); 2 School of Built Environment, The University of New South Wales, Sydney, NSW 2052, Australia; s.zlatanova@unsw.edu.au; 3 Department of Civil Engineering, Toronto Metropolitan University, 350 Victoria Street, Toronto, ON M5B 2K3, Canada; snli@torontomu.ca

**Keywords:** smartphone indoor positioning, multi-source sensors, fusion-based positioning methods, indoor location services

## Abstract

Positioning information greatly enhances the convenience of people’s lives and the efficiency of societal operations. However, due to the impact of complex indoor environments, GNSS signals suffer from multipath effects, blockages, and attenuation, making it difficult to provide reliable positioning services indoors. Smartphone indoor positioning and navigation is a crucial technology for enabling indoor location services. Nevertheless, relying solely on a single positioning technique can hardly achieve accurate indoor localization. We reviewed several main methods for indoor positioning using smartphone sensors, including Wi-Fi, Bluetooth, cameras, microphones, inertial sensors, and others. Among these, wireless medium-based positioning methods are prone to interference from signals and obstacles in the indoor environment, while inertial sensors are limited by error accumulation. The fusion of multi-source sensors in complex indoor scenarios benefits from the complementary advantages of various sensors and has become a research hotspot in the field of pervasive indoor localization applications for smartphones. In this paper, we extensively review the current mainstream sensors and indoor positioning methods for smartphone multi-source sensor fusion. We summarize the recent research progress in this domain along with the characteristics of the relevant techniques and applicable scenarios. Finally, we collate and organize the key issues and technological outlooks of this field.

## 1. Introduction

In recent years, the rapid development of smartphone technology and the large-scale deployment of the fifth-generation mobile communication network (5G) have greatly boosted the development of smart mobile devices and the mobile Internet services [1], and the indoor location navigation service industry based on smartphones has developed rapidly. Nowadays, with people spending over 80% to 90% of their time indoors [2], the demand for indoor location services is increasing due to the development of smartphones and smart cities [3]. At the same time, with the acceleration of urbanization, large shopping malls, airports, railway stations, and other large and complex building complexes continue to emerge. The demand for location-based services (LBS) has witnessed a significant shift from outdoor to indoor environments, with increasing sectors such as transportation, medical care, and emergency monitoring expressing a strong need for indoor location services [4]. While the Global Navigation Satellite System (GNSS) is the most widely used positioning tool outdoors, for indoor environments, there remains a lack of standardized technology and software interfaces for indoor environments that would enable devices to self-localize or be localized using existing infrastructure [5]. Due to the widespread adoption of diverse technologies such as Wi-Fi, 5G, Bluetooth Low Energy (BLE), ultra-wideband (UWB), radio frequency identification (RFID), and others, future networks will exhibit a high degree of heterogeneity [6]. The possible coexistence of a heterogeneous indoor localization system (ILS) gives rise to the problem of switching from an ILS to a different one [7]. This poses significant challenges regarding the need for a proper integrated architecture and standardization in ILSs. Furthermore, GNSS positioning technology is unable to provide reliable positioning services in indoor environments due to signal occlusion [8], the multipath effect [9], and the attenuation [10] of satellite signals in indoor environments, resulting in challenges for pedestrians to accurately determine their current location and navigate within buildings. Therefore, as one of the most important parts of indoor location-based services, the development of indoor positioning technology for smartphones with high availability, high accuracy, robust functionality, and low cost has become the key to the realization of seamless location-based services (SLBS) and Internet of Things (IoT) applications [11].

With the increasing abundance of sensors in smartphones, smartphone-based localization has become more convenient and efficient. In recent years, a large number of indoor localization techniques have been explored, mainly including wireless techniques such as BLE [12,13], Wi-Fi [14], magnetic field [15], 5G [16], foot-mounted ultrasonic sensors [17], acoustic [18], UWB [19,20], visible light [21], RFID [22], Light Detection and Ranging (LiDAR) [23]; and relative positioning-based techniques such as the inertial navigation system (INS) [24], the strapdown inertial navigation system (SINS) [25], pedestrian dead reckoning (PDR) [26], or Quick Response (QR) code positioning [27], which are realized by collecting data from several built-in sensors of smartphones, such as accelerometers, magnetometers, gyroscopes, and QR markers, to achieve indoor localization.

**Figure 1:**
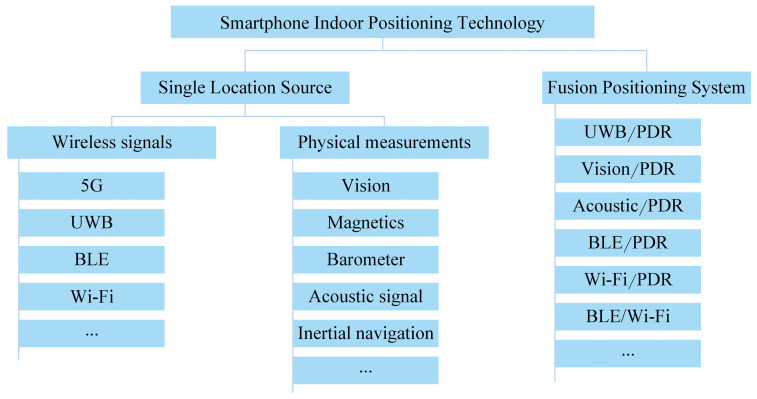
Overview of smartphone indoor positioning methods.

An overview of smartphone indoor positioning technologies is shown in Figure 1. However, there is a lack of general-purpose technologies like GNSS. Each method has advantages and disadvantages, and there is no single technology that prevails in all practical scenarios regarding accuracy, power consumption, and portability. Universality and deployment cost are the key factors determining each localization method’s applicability. Wi-Fi has wide coverage and low cost, but the signal is susceptible to interference and blockage, and the creation and maintenance of fingerprint databases are very tedious tasks. Some map-based methods can improve the localization [28], but such methods are tested only in lab environments. BLE-based solutions are widely adopted due to their superior performance in terms of cost-effectiveness, high accuracy, and ease of deployment; however, the coverage range is limited. The ultrasonic foot-mounted sensors provide accurate localization but can require additional equipment restricting the range of users. The vision-based indoor positioning technology offers several advantages, including the elimination of base station deployment requirements, immunity to signal strength variations affecting positioning accuracy, and relatively low operational costs. However, the lighting conditions of indoor environments, sparse recognizable elements, and background interference all impact the positioning results, and appropriate processing and correction are required. The UWB method has high anti-jamming and penetration capabilities and can achieve centimeter-level positioning accuracy. Still, the high deployment cost of the technology remains a significant barrier to its widespread adoption. The PDR technique, widely employed for indoor localization, offers the advantages of low computational load and continuous localization, and it does not require the deployment of additional equipment to complete the localization work. However, PDR is susceptible to error accumulation, leading to a decrease in positioning accuracy over time. QR markers are a very low-cost solution, but they require users to actively look for available markers to scan them.

Currently, the mainstream indoor positioning technologies have their specific application scenarios. After considering the actual project implementation cost, accuracy, efficiency, and other relevant factors, it is evident that there is no universally applicable indoor positioning solution for smartphones. In contrast, the multi-source sensor fusion localization technology is the current research hotspot, which facilitates the complementary advantages between different sensor localization technologies [29].

In recent years, scholars have proposed to integrate two or more indoor positioning techniques into a new one, which can achieve better positioning performance by leveraging the advantages of different methods. Approaches based on the built-in sensors of smartphones are usually combined with different positioning sources, such as Wi-Fi or BLE, to realize the complementary advantages of different sensor positioning modules and adapt to complex and changing indoor environments.

### 1.1. Existing Related Surveys

This review complements already existing reviews on indoor positioning using smartphones with multi-source sensor fusion. Morar et al. (2020) [30] and Kunhoth et al. (2020) [31] reviewed computer vision-based indoor localization methods including advantages and disadvantages, navigation, and computer vision-based localization systems. Liu et al. (2020) reviewed distance-based acoustic indoor localization divided into absolute distance localization and relative distance localization [32]. Guo et al. (2020) proposed a fusion-based indoor localization technique and system with three fusion features: source, algorithm, and weight space [33]. However, this is only an extensive summary and analysis of indoor positioning. Liu et al. (2020) reviewed the existing Radio Frequency (RF)-based indoor positioning systems in terms of principles, techniques, and system architecture [34]. Ashraf et al. (2020) reviewed the methods for estimating the user’s indoor location by using the data from smartphone sensors and discussed the challenges associated with these methods by analyzing the methods on smartphone sensors [35]. However, there is less discussion on methods that utilize multiple sensors for fused localization. Simões et al. (2020) conducted a study on indoor navigation and localization systems. However, the focus of this research is on the blind [36]. Pascacio et al. (2021) provide a systematic review of collaborative indoor positioning systems, identifying several promising future research avenues and research gaps based on the analysis and results [37]. Obeidat et al. (2021) provide a review of indoor localization techniques and wireless technologies, and provide a detailed account of localization detection techniques and commonly used localization algorithms and methods, but do not discuss methods for fusing multiple localization sources [38]. Hou and Bergmann (2021) provide a systematic review and a quality assessment of the research on PDR with wearable sensors. However, they only focused on the computer vision area [39]. Ouyang and Abed-Meraim (2022) conducted a review based on magnetic fingerprinting localization techniques used in indoor environments and also summarized the magnetic field calibration algorithms [40]. Aparicio et al. (2022) classified acoustic positioning systems into different groups and summarized the main characteristics of these systems in terms of accuracy, coverage area, and update rate [41]. Wang et al. (2022) provide a systematic and in-depth review of smartphone-based inertial localization and navigation methods that have emerged in recent years, providing the reader or researcher with a complete view of the pedestrian inertial navigation field [42]. However, this study mainly discusses inertial navigation. Chen and Pan (2024) reviewed related work on deep learning-based inertial localization, describing how deep learning can be applied to sensor calibration, localization error drift reduction, and sensor fusion [43]. Naser et al. (2023) systematically combed through and analyzed indoor localization methods based on smartphones, according to the smartphone inclusion and exclusion criteria to select articles, elaborated on the effects of pedestrian movements and phone poses, and also analyzed the motivations and challenges of using smartphones for indoor positioning [44]. However, they focused on the motivations and challenges of indoor localization with smartphones rather than elaborating on each localization method. Zhuang et al. (2023) provided an extensive review of combined multi-sensor navigation/localization systems, categorized fused localization systems based on localization sources, algorithms and architectures, and scenarios, classified the systems as analysis-based fusion or learning-based fusion, and analyzed them in detail [45]. We compare the latest and most relevant surveys in Table 1.

### 1.2. Key Contributions

Numerous recently existing review studies provide a detailed overview of the fundamentals and technical guidance for a particular indoor positioning technique, while there are fewer review papers that detail smartphone indoor positioning systems based on a single positioning technique as well as smartphone indoor positioning systems with multi-source sensor fusion. Although there have been many studies focusing on smartphone-based indoor localization methods, the performance, advantages, and disadvantages of these methods still need to be systematically summarized and analyzed, and these methods still face challenges that need to be addressed. This paper reviews the research results of indoor positioning based on smartphone sensors, classifies these results and analyzes the current mainstream multi-source sensor fusion smartphone indoor positioning methods, and summarizes their basic principles and technical characteristics as well as the advantages and key issues of these methods. In addition, we discuss utilization trends and provide practical suggestions for researchers and developers to help them select and improve indoor localization methods in practical applications.

The main contributions of this work are listed as follows.

1. In this study, we detail the current research status of smartphone-based indoor positioning methods, categorize and analyze the characteristics of commonly used indoor positioning techniques, and categorize and describe these techniques. By reviewing the existing literature, we summarize the basic principles, advantages, and limitations of various techniques.
2. Based on the mainstream indoor positioning technologies on smartphones, we conducted a detailed review and analyzed the existing indoor positioning methods and the main features of the latest research for each technology. Each technology is summarized and its performance in terms of indoor positioning performance is analyzed.
3. We examine and classify the indoor positioning methods commonly used for fusing multi-source sensors in recent years, introduce the principles and characteristics of each fusion method, and analyze and summarize the related articles in recent years. We discuss how the fusion methods can improve the positioning accuracy and robustness as well as the limitations.
4. We examine and classify smartphone-based single-sensor localization techniques and multi-source sensor fusion localization techniques. Based on these challenges, we suggest directions for future optimization of indoor positioning techniques.

## 2. Smartphone Indoor Positioning Methods

An overview of indoor localization methods with a single source is shown in Table 2, where the comparison indicates that the single sensor localization methods have their advantages and disadvantages in different scenarios. In Table 2’s accuracy section, it is noted that the inertial navigation system suffers from error accumulation. The initial positioning accuracy is high, but as the positioning time increases, the error rapidly grows. For the acoustic sensor-based system, it is more sensitive to the Doppler effect caused by the motion between transmitters and receivers. Therefore, deploying more infrastructure can improve the system’s positioning accuracy. Moreover, in the cost section of Table 2, the considerations include the procurement and deployment costs of equipment required for the systems, as well as the human and material resources necessary to maintain the systems. Therefore, the integration of multiple sensor sources in indoor positioning and navigation methods for smartphones enhance positioning accuracy through the coordination of ranging information from diverse sensors, thereby mitigating their respective limitations and capitalizing on their individual advantages [46].

**Figure 2:**
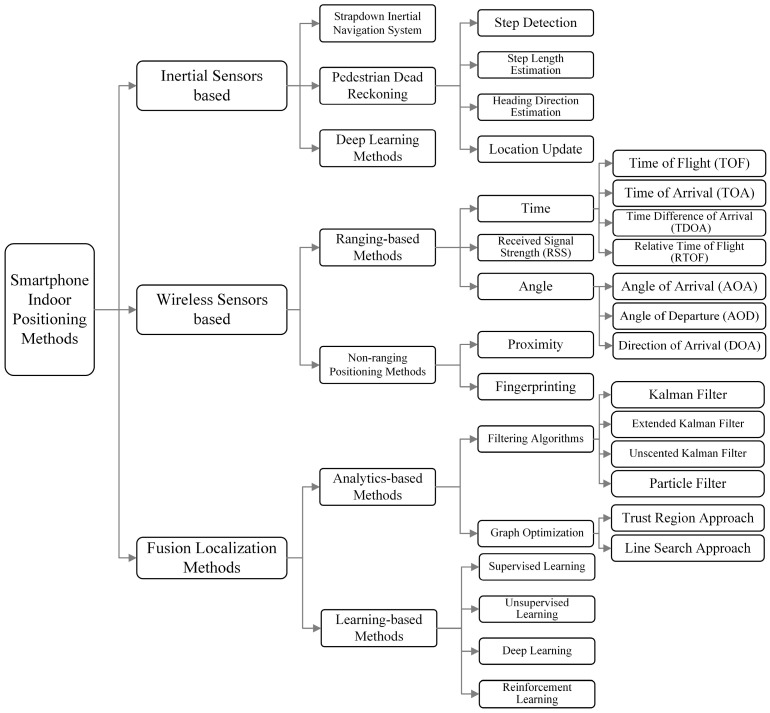
Classification of smartphone indoor positioning methods.

With the continuous development of smartphone sensors and their performance, the pervasive localization methods based on the built-in sensors of smartphones have become one of the main research directions, and fusing multi-source sensors has gradually become a research hotspot. The current classification of mainstream smartphone indoor positioning and navigation technologies is shown in Figure 2. Smartphones have a variety of sensors that can be used as data sources for indoor positioning, such as Wi-Fi, BLE, UWB, 5G, acoustic signals, accelerometers, gyroscopes, barometers, and cameras.

### 2.1. Single Sensor-Based Indoor Localization Method for Smartphones

#### 2.1.1. Wi-Fi-Based Indoor Localization Method

Wi-Fi is a wireless networking technology that allows electronic devices to communicate with each other without the need for direct physical connections. Wi-Fi technology follows a variety of IEEE 802.11-standard protocols to ensure a wide range of compatibility and performance levels. In indoor positioning, Wi-Fi determines location by analyzing the signal strength or time of flight between the device and multiple access points, enabling smartphone-based indoor location applications such as mall navigation, asset tracking, and pedestrian location-based navigation. Common measurements in Wi-Fi positioning systems include the time of flight (TOF), angle of arrival (AOA), direction of arrival (DOA), channel state information (CSI) [47], and received signal strength indicator (RSSI). The commonly used Wi-Fi localization methods can be categorized into fingerprint-based localization methods [48] and RSSI-based localization methods. The fingerprint-based method collects Wi-Fi RSSI features from each point in the localization area to form a Wi-Fi fingerprint database. Then the Wi-Fi RSSI features collected online are matched with the database to obtain the pedestrian location. There are two schemes for Wi-Fi RSSI-based localization methods. The first method uses a Wi-Fi RSSI attenuation model to calculate the distance between the mobile device and the Wi-Fi AP. The second one is based on TOF ranging, which is exclusively supported by the extension of the 802.11mc standard, also known as Wi-Fi Round-Trip Time (RTT). This standard is the only certified extension by the Wi-Fi Alliance that uses the Fine Time Measurement (FTM) protocol for precise distance measurement. It enables devices supporting this protocol to determine their location based on the time it takes for a wireless signal to travel to an AP and back. In contrast, AOA and DOA techniques are defined within the 802.11az standard, referred to as Wi-Fi next-generation positioning. This standard aims to enhance positioning accuracy but is not yet supported by the FTM protocol. Table 3 summarizes the characteristics of different Wi-Fi-based indoor localization methods.

Wang et al. (2017) applied the fingerprint recognition method to CSI-based indoor localization, using an Intel 5300 card to receive the CSI phase information, and achieved better localization accuracy [52]. Xu et al. (2018) presented a pedestrian tracking algorithm that integrates environmental constraints from an indoor grid model, Wi-Fi positioning technology, and a mobile device’s magnetometer, significantly improving localization accuracy and reducing tracking errors [28]. Amri et al. (2019) proposed a new localization algorithm that uses RSSI as an input to the fuzzy inference system and calculates the distance between the anchor point and the sensor node through the channel flow, which was proved to be energy efficient and effective by experimental results [49]. To address the non-line of sight (NLOS) and multipath problems faced by indoor localization based on radio signals, Huang et al. (2020) designed a deep convolutional neural network to learn the nonlinear mapping relationship between indoor spatial location and Wi-Fi RTT ranging [57]. Yu et al. (2020) analyzed the impact of Wi-Fi FTM ranging accuracy and proposed corresponding calibration, filtering, and modeling algorithms that can effectively reduce the ranging errors caused by clock bias, NLOS, and multipath propagation [58]. Guo et al. (2024) proposed a monitoring-based localization system using a theoretical model of clock drift to reduce the impact of clock drift on indoor ranging and localization results of Wi-Fi RTT [59]. The algorithm proposed by Vishwakarma et al. (2023) utilizes a graph neural network to accurately categorize a specific location into its corresponding region based on the collected RSSI values, thereby enhancing the precision of location prediction [51]. To solve the problem of substandard quality in Wi-Fi indoor localization fingerprint database constructed by traditional methods, Pan et al. (2024) proposed an indoor localization fingerprint database construction method based on the crow search algorithm optimized density-based spatial clustering of applications with noise and recurrent conditional variational autoencoder-generative adversarial network. This method requires only a sparse collection of reference point location coordinates and RSS data, enabling the construction of a high-quality indoor Wi-Fi localization fingerprint database and improving the localization capability [56]. Cao et al. (2024) improved the indoor localization accuracy using the Wi-Fi RTT algorithm, which was able to compensate for LOS localization errors and identify NLOS conditions with an average absolute error of 1.08 m [60]. To address the issue of significant variations in Wi-Fi technology’s localization performance across different indoor environments, Feng et al. (2024) used a machine learning weighted model to dynamically select the optimal Wi-Fi localization model for each location [61].

The Wi-Fi-based indoor localization offers the advantages of cost-effectiveness, simplified maintenance, and extensive coverage, making it a more suitable approach for wide-area targets in indoor localization. However, Wi-Fi-based indoor positioning methods still face several challenges. Wi-Fi fingerprint localization is time-consuming and inefficient, and establishing and updating the fingerprint database is tedious. The localization performance heavily relies on how frequently and accurately the database is updated. Wi-Fi RSSI ranging and localization is susceptible to factors such as multipath, obstacles, and non-ranging signals, which limits its accuracy in complex indoor environments. The Wi-Fi RTT ranging technique can achieve meter-level accuracy but requires specific device support and exhibits potential performance degradation in high-temperature, high-traffic scenarios. Therefore, in more in-depth research, Wi-Fi-based indoor positioning methods need to further optimize the updating methods of fingerprint databases to reduce the human and time costs associated with data updates; enhance anti-interference capabilities to improve stability and reliability; and integrate other positioning technologies, such as Bluetooth and PDR, to achieve higher accuracy.

#### 2.1.2. Bluetooth-Based Indoor Localization Method

Bluetooth is also a widely used wireless communication technology. Bluetooth beacons are extensively embraced by smart devices due to their compact size, lightweight design, cost-effectiveness, and energy efficiency. In indoor positioning, compared to Wi-Fi, Bluetooth Low Energy (BLE) consumes less energy and offers superior support for smart devices, making it a more flexible and easily deployable option. Based on this technology, Bluetooth-based smartphone indoor positioning methods have become widely adopted, particularly for pedestrian indoor navigation, employee activity tracking, and providing visitors with turn-by-turn navigation in large venues like airports or malls. Bluetooth localization can be categorized into fingerprint-based methods [62], proximity detection [13,63], and ranging-based methods. The localization accuracy of fingerprint-based methods generally depends on the refinement of the fingerprint database construction. Proximity detection determines the relative proximity of the device to the signal source by detecting the signal strength of the device and the Bluetooth beacon. This helps determine which area or reference point the device is roughly located in. The accuracy of this method is relatively low but can be improved by complementing with semantic maps to eliminate impossible locations. The ranging-based method calculates the distance between the beacon and the Bluetooth AP by deploying a certain amount of Bluetooth beacons in indoor environments, measuring the RSSI at the receiving end through the pre-built signal propagation loss model (SPLM), and solving the geometric relationship with planar trilateral localization or spatial quadrilateral localization algorithms. In addition, the study of BLE AOA [64] has led to the opening of new possibilities for BLE localization based on BLE goniometry. Table 4 summarizes the characteristics of different Bluetooth-based indoor localization methods.

Spachos et al. (2020) proposed an indoor positioning system relying on the proximity and localization capabilities of BLE beacons to enhance the user experience in museums by automatically providing users with cultural content related to the observation of artifacts [73]. You et al. (2021) analyzed the effect of pedestrian swinging arms on the performance of the PDR and used RSSI-based multipoint positioning algorithms to reduce the cumulative error [65]. Yu et al. (2023) proposed an intelligent three-dimensional (3D) indoor localization framework integrating Wi-Fi, BLE, QR code, and inertial measurement unit (IMU) sensors, which can achieve autonomous and accurate localization in large indoor areas using different location sources [74]. Assayag et al. (2023) improved the estimation of BLE signal strength distance by adaptively selecting the optimal parameters of a logarithmic distance model, which reduces the average error compared to a fixed parameter approach [67]. Safwat et al. (2023) used KNN and WKNN to match the collected RSSI readings with the RSSI of an unknown location in order to determine the user’s location and were able to obtain small localization errors even under obstacles, reflections, and interference conditions [69]. Wu et al. (2024) addressed the problem of high Bluetooth signal fluctuation and significant localization error during indoor navigation by using the Kalman filter (KF) to attenuate the effect of random perturbations on the true Bluetooth signal, utilizing maximum likelihood estimation to infer the pedestrian’s positional coordinates, and using multiple beacon nodes to improve the localization accuracy [68].

Bluetooth positioning method provides different levels of accuracy (from room level to centimeter level), which makes it widely applicable to different scenarios. The Bluetooth positioning technology exhibits low cost, low power consumption, ease of deployment, compact device size, and widespread availability in mobile phone terminals equipped with Bluetooth modules, and it is easy to widely popularize and implement on a large scale. However, it also has similar limitations to Wi-Fi, such as high manual costs for building fingerprint databases, and performance degradation due to multipath effects, signal blockage, and RSSI fluctuations. In addition, this technology is closely associated with beacon deployment and is limited by a small range of action susceptible to external noise signal interference, resulting in poor signal stability. Bluetooth-based indoor positioning methods need to explore directions such as enhancing signal processing techniques to mitigate issues caused by multipath effects and signal interference. Additionally, optimizing the placement algorithms for Bluetooth beacons is crucial; this would ensure that coverage is maximized while maintaining sufficient signal strength to support high-precision positioning.

#### 2.1.3. Inertial Sensors-Based Indoor Localization Method

In contrast to other localization strategies, inertial navigation algorithms use motion information measured by inertial sensors to estimate the pedestrian’s position relative to the starting point. INS is a completely independent method of localization and navigation that estimates attitude, velocity, and position without any dedicated infrastructure or pre-trained fingerprints. Therefore, INS has no coverage limitations and can work anywhere [75].

With the rapid development of sensor technology, low-power and low-cost inertial measurement units (IMUs) are widely used in portable smart devices. The inherent advantages of smartphones as portable communication devices (built-in various sensors with powerful computing and storage capabilities) make smartphones a prominent platform for indoor positioning and navigation [76]. The obvious advantage over other indoor positioning platforms is that users only rely on smartphones they carry and do not need additional hardware to provide indoor positioning services.

##### Accelerometer

Accelerometers are sensors used to measure the acceleration of an object by using the inertial force generated during its motion to find the acceleration of the object at the current moment, which can be used to measure the motion, attitude, vibration, and other information of the object. Nowadays, smartphones usually have built-in triaxial accelerometers, which are used to detect the magnitude and direction of the acceleration that the smartphone is subjected to. Three-axis accelerometers can measure the specific force of an object, which is the overall acceleration after removing gravity, while also enabling angle measurements. Due to its working principle of measuring angles, the three-axis accelerometer cannot measure yaw angles but can measure pitch and roll angles. In indoor positioning applications, the accelerometer is mainly used to detect the gait and movement speed of pedestrians, and in conjunction with other inertial sensors, to collaborate on position localization and updating. In addition, it can capture several typical motion patterns of a smartphone such as shaking and flipping, so that the smartphone can be utilized to sense the motion state. However, the accelerometer is susceptible to error accumulation, leading to an increasing distance error as the positioning time prolongs.

Jeon et al. (2015) proposed an indoor pedestrian localization system based on accelerometers, barometers, and Bluetooth RSSI to estimate the walking distance of pedestrians by obtaining the data of the phone from the accelerometer on the smartphone [77]. Lee et al. (2018) proposed an indoor localization system using BLE, accelerometers, magnetometers, and barometers of a smartphone. Accelerometers and magnetometers were utilized to locate the indoor pedestrian’s movement position. The experimental results show that the method has good localization performance [78]. Yan et al. (2022) integrated the measurement of multiple multi-module BLE and the measurement of smartphone accelerometers. The unscented Kalman filter (UKF) method used to estimate position and velocity was constrained using the pedestrian walking speed obtained from the accelerometer and a better localization accuracy was obtained [79].

##### Gyroscope

Gyroscopes, also known as angular velocity sensors, are used to detect the angular velocity of a carrier and to calculate the angle after integrating the angular velocity. Gyroscopes are capable of measuring angular velocity along one or several axes of motion, enabling the quantification of a device’s turn or grade change; however, they are also susceptible to cumulative errors. The physical parameters of a gyroscope include sensitivity, zero bias, noise, temperature drift, stability, non-orthogonal error and scale factor error. The three-axis gyroscope is the core sensitive device of the inertial navigation system, and its measurement accuracy directly affects the accuracy of the attitude solution of the inertial navigation system. In indoor positioning applications, the built-in three-axis gyroscope sensor of smartphone can detect a phone’s movement direction, identify the attitude information, and obtain the position information related to the movement of the pedestrians. Gyroscopes and accelerometers can combine their respective advantages. By using both an accelerometer and gyroscope, it is possible to better track and capture the complete motion in 3D space, providing a more accurate navigation system.

Bai et al. (2020) achieved higher localization accuracy using an adaptive error compensation algorithm based on motion velocity and step detection based on up and down tracking when using only gyroscopes and accelerometers [80]. Chen et al. (2023) proposed a trajectory matching method based on the hidden Markov model (HMM), which utilizes gyroscope and accelerometer sensor sequences that are not sensitive to the initial motion state of the attitude changes and trajectory length are estimated and modeled, and the initial motion is inferred and the trajectory is refined based on spatial information [81].

##### Magnetometer

The magnetometer is capable of detecting the strength of the magnetic field to which the equipment is subjected in the X, Y, and Z axes, thus determining the heading angle in relation to the geomagnetic north pole. Magnetometers can measure the strength and direction of the magnetic field at different locations within a building and can help eliminate errors in gyroscope readings. The natural existence of the geomagnetic field is contrasted with the indoor geomagnetic field, which is influenced by building structures and large electronic equipment, resulting in changes to its magnetic strength signal characteristics. By utilizing fingerprinting positioning methods based on these distinct indoor magnetic field features, magnetometers can accurately estimate location.

Ashraf et al. (2020) proposed the construction of a database for geomagnetic field patterns utilizing data collected by magnetometers integrated into multiple smartphones. The results showed that the localization performance of four different smartphones was almost the same and overall improved [82]. Kuang et al. (2022) used the relative trajectory and attitude generated by the PDR for magnetic field matching and used the attitude information to project the reference magnetic field map from the navigation coordinate system to the body coordinate system to eliminate the effect of magnetometer bias [83]. Qadr et al. (2022) proposed an indoor localization technique using the combination of Wi-Fi signals and magnetometers to minimize errors and improve the positioning of smartphones [84].

When solely relying on a magnetometer, the absence of horizontal sensor placement verification may result in significant deviations in the measured heading angle. Additionally, the magnetometer is susceptible to distortion caused by interference from the indoor magnetic field environment. Therefore, the tilt angle of the magnetometer is usually corrected using acceleration. The corrected magnetometer can be used in combination with a gyroscope for attitude estimation, and the drift error of the gyroscope can be corrected by the heading angle measured by the magnetometer. However, when there is a sudden change in the magnetic field around the magnetometer, such as the appearance of magnetic substances or an increase in current, it can cause distortion of the magnetometer.

##### Inertial Sensor Fusion for Indoor Localization

With the development of micro-electromechanical systems (MEMS) technology, MEMS-based IMU are embedded in most smartphones and tablet devices due to their cost-effectiveness, compact size, and energy efficiency [85]. Smartphones are equipped with several sensors to detect walking patterns and behaviors [86]. Walking patterns represent the overall movement of a pedestrian, including walking, running, going up or down stairs, and using elevators or escalators. Data collected by inertial sensors included in smartphones can identify the activities performed by the person holding the device [87], and these data can be used to improve localization accuracy [88].

**Figure 3:**
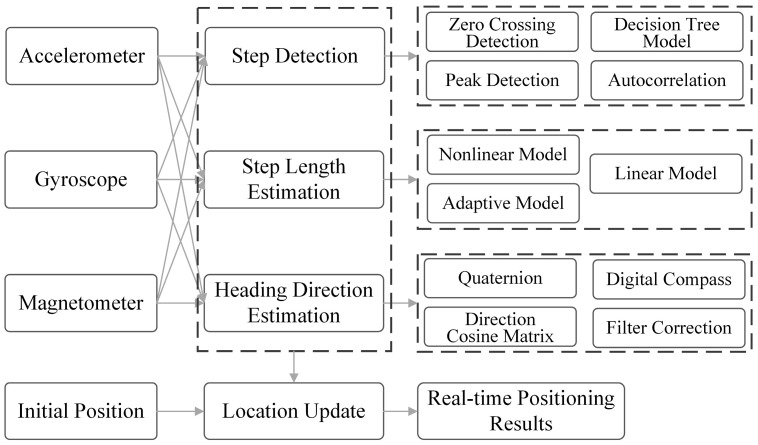
PDR positioning methodological framework.

At present, there are mainly three methods of pedestrian positioning based on smartphone IMU sensors. The first approach is based on the conventional inertial navigation solution, wherein the three-axis acceleration of the IMU is utilized to perform double integrations for deriving 3D velocity and position. Additionally, angular velocity integration is employed to determine attitude, while fitting the displacement curve. However, in practical applications, due to the limitation of device accuracy and the existence of integral accumulation error, its calculation error often reaches tens of meters or even hundreds of meters in just a few seconds. The second approach is IMU sensor-based PDR, which leverages the acceleration and angular velocity data captured by the IMU sensor to conduct gait analysis, estimate step count and length, and extrapolate pedestrian’s position, attitude, and other relevant information by integrating with heading data obtained from angular velocity integration. The framework of the PDR localization method is shown in Figure 3. The PDR localization method usually consists of four basic phases: step detection, step length estimation, heading estimation, and position update. For step detection, various algorithms have been proposed in recent years, such as zero crossing detection [89], peak detection [90], pitch angle detection [91], the autocorrelation method [92], and the decision tree model [93]. Step length estimation models include linear, nonlinear, and adaptive models. Common heading estimation methods include quaternion [94], digital compass [95], direction cosine matrix, and filter fusion. The localization accuracy of PDR mainly depends on the accurate estimation of step length, step frequency, and heading angle. Consequently, as walking time increases, the localization error accumulates due to the cumulative errors in inertial devices. Another method is the SINS, through which the real-time 3D attitude and position can be obtained. SINS mounts one or more IMUs devices to the human body (foot, legs, waist, and so on), leverages the INS mechanization algorithm to estimate pedestrian displacement, and utilizes zero-velocity update (ZUPT) to reset integral errors [96]. However, mounting dedicated devices on the human body not only increases costs but also increases the additional burden. Since there is no zero-velocity status for handheld smartphones, few works identify quasi-static to replace zero velocity and try to migrate the INS mechanization algorithm to the smartphone.

Although the performance of the PDR algorithm is limited by the cumulative errors of low-cost sensors, complex hand-held and motion patterns, and indoor magnetic interference, it remains the predominant solution for pedestrian indoor localization and navigation. Moreover, in the field of pedestrian indoor localization and navigation, its accuracy is still much better than the traditional inertial guidance solving method. Consequently, it has received considerable attention from researchers and scholars who continuously strive to enhance its capabilities (Table 5).

Klein et al. (2018) used a machine learning approach to classify variable handheld modes of mobile devices and improved the accuracy of PDR by choosing appropriate gain values [97]. Guo et al. (2019) proposed a pedestrian walking speed estimation algorithm considering four different handheld modes of a smartphone, which tightly integrated the classified handheld modes using adaptive gait recognition. The final experimental results showed that the classification accuracy of the framework was 98.85%, and the speed estimation error was less than 0.06 m/s [98]. Zheng et al. (2020) investigated the pocket and swing modes of a smartphone and analyzed the relationship between rotational motion, walking state, and heading information. An improved rotation method and a single-point algorithm were proposed for heading estimation in pocket mode and swing mode, respectively, which effectively improved the final performance [99]. Yao et al. (2020) combined zero crossing detection with dynamic time-warping-based peak prediction to accurately identify the start and end points of each step in the walking pattern [100]. Zhang et al. (2021) proposed a method with a cascade filtering structure. The upper PF is fused with the map information to correct the estimation results of the lower filter, thus correcting the navigation error and improving the positioning accuracy [101]. Zhao et al. (2023) designed a two-feature step detection model to optimize the step length estimation and heading estimation and proposed an adaptive model to identify different walking states to correct the rapid accumulation of the error in the calculation of pedestrian trajectories [102]. Wu et al. (2024) proposed a multi-sensor fusion indoor PDR algorithm to improve and integrate the step detection, step length estimation, and heading estimation algorithms, which effectively reduces the error accumulation [103]. To improve the performance of PDR, Liu et al. (2024) proposed an enhanced PDR algorithm that integrates pedestrian motion constraints, smartphone sensors, and a combined step detection method to deliver a continuous, precise, and dependable localization solution [104]. Chen et al. (2024) introduced a 3D localization method based on terrain feature matching to identify pedestrian movement patterns and effectively match the corresponding location coordinates, thereby mitigating the impact of cumulative errors in multi-floor scenes [105].

The advantages of using inertial sensors for indoor localization are their independence (work without relying on external infrastructures), ease of integration, lack of interference from indoor wireless signals, and cost-effectiveness. PDR algorithms, extensively employed in indoor localization, however, exhibit a cumulative error over time, resulting in gradual deviation of the position estimation from the actual path as well as a substantial reliance on sensor quality and algorithm design. The accuracy of PDR systems has been significantly improved through the application of algorithm optimization, filtering techniques, and machine learning methods. However, as a relative localization technique, inertial sensors typically rely on other sources of localization to provide initial position coordinates and correct PDR results in order to minimize accumulated error.

#### 2.1.4. Barometer-Based Indoor Localization Method

Barometer is a sensor used to measure atmospheric pressure, which is affected by parameters such as the temperature, weather, and humidity of the environment. In indoor positioning, barometers can be used to measure changes in indoor air pressure to infer the location and height of a device. Although the barometer sensor cannot be positioned as a stand-alone positioning sensor, it is often used to determine the change in altitude when the PDR is positioned, and it can measure atmospheric pressure to measure altitude using the elevation equation [106]. Barometers are used to monitor vertical movement, and changes in atmospheric pressure as the user moves up or down help determine changes in altitude [107]. As the user moves, the elevation change can help in localizing the user to a particular floor.

Xu et al. (2017) proposed a barometer-based floor localization algorithm, which calculates the height change through barometer measurements and introduces a Bayesian network model to infer the change of the floor where the pedestrians are located [108]. Elbakly et al. (2018) proposed a multi-sensor fusion floor localization system that combines barometer sensors and Wi-Fi APs mounted in buildings into a probabilistic framework to identify the floor where a pedestrian is located [109].

Integrating barometers into smartphone-based indoor positioning still faces several challenges. Barometers are susceptible to external influences, such as temperature, humidity, and wind, leading to varying readings throughout the day. Accurate altitude determination requires initial calibration against a known reference point; without this, accumulated errors can degrade positioning performance. In addition, while barometers provide altitude information, their resolution may not be sufficient for distinguishing between closely spaced floors in multi-story buildings, limiting vertical positioning accuracy. These limitations necessitate integrating barometers with other methods for more practical outcomes.

#### 2.1.5. Vision-Based Indoor Localization Method

The smartphone camera refers to a camera module integrated into a smartphone device, which is utilized for capturing photographs and recording videos. Typically, it comprises one or multiple lenses, image sensors, image processing chips, and other associated components. The visual positioning system (VPS) utilizes smartphone camera sensors to capture visual features of the indoor environment and uses them to estimate the location of pedestrians. In indoor localization, vision-based indoor positioning methods are constantly evolving and can be broadly classified into three categories. The first category of methods detects objects in an image and matches them with objects in a building database. The second category is image-based visual localization methods. Accurate estimation and localization of indoor locations is achieved by analyzing image or video data in the scene, extracting features and matching them with a pre-constructed reference map. The last category utilizes deployed visual markers, including concentric circles, barcodes, QR codes, or special patterns.

In recent years, advancements in mobile SLAM technology have significantly influenced vision-based indoor localization. Mobile SLAM algorithms enable smartphones to simultaneously create a map of an unknown environment while keeping track of their position within it. This technology is crucial for applications such as augmented reality (AR), indoor location and navigation, and autonomous systems. Modern smartphones leverage powerful processors and specialized hardware accelerators to run SLAM algorithms efficiently, providing real-time tracking and mapping capabilities. Several commercial software development kits (SDKs) facilitate the integration of SLAM and other AR functionalities into mobile applications [110]. Notably, Google’s ARCore excels in environmental understanding through motion tracking, environmental lighting estimation, and depth perception using the phone’s camera and sensors. Apple’s ARKit offers robust plane detection, object occlusion, and accurate motion tracking by combining visual inertial odometry with data from the accelerometer and gyroscope. Huawei’s AREngine provides similar features tailored for its devices, focusing on optimizing performance and user experience. Wikitude is a cross-platform AR SDK supporting iOS, Android, and other devices, renowned for its powerful location-based and instantaneous tracking capabilities. Besides these, Microsoft’s Mixed Reality Toolkit (MRTK) supports development for mixed reality applications across multiple platforms, integrating spatial mapping and spatial understanding. These SDKs provide developers with tools and application programming interfaces (APIs) that simplify the implementation of complex computer vision tasks. They also support advanced features like plane detection, object recognition, and image anchors, which enhance the accuracy and robustness of indoor localization solutions. In addition to AR-related SDKs, there are also other development toolkits that use vision and other types of sensors, such as the Intel RealSense SDK and Bosch Sensortec Sensor API. These SDKs can leverage data fusion from different types of sensors to achieve complex functionalities like precise positioning, environmental understanding, and user behavior analysis. There have been many studies on visual-based localization methods, as shown in Table 6.

Xiao et al. (2018) proposed an indoor localization system based on computer vision, which utilized static objects in the room as a reference for estimating the user’s position to achieve a localization accuracy of up to 1 m at a low cost [114]. Tanaka (2020) developed an ultra-high precision visual marker with an attitude estimation error of less than 0.1°, which effectively reduces the reprojection error and decreases the position error of the marker by using the good attitude accuracy of his proposed method [117]. Kubícková et al. (2020) utilized the SIFT and the PnP problems to compute the camera pose and position for the indoor localization of pedestrians [112]. Li et al. (2021) classified different scenes based on a deep confidence network and used the PnP algorithm to extract spatial reference points from deep images to solve for the camera position [113]. Jung et al. (2021) utilized both point cloud and RGB feature information to accurately acquire indoor 3D space. They estimated the indoor 3D space using a modified point map neural network that incorporated improved target pose information, which was then matched with the pose information labels of a pre-constructed voxel database to determine the user’s position [115]. Xu et al. (2021) present an infrastructure-free indoor navigation system that guides future users by reusing the trajectory experience of previous travelers in a peer-to-peer mode, leveraging visual SLAM technology on commercial smartphones and optimizing for robustness against environmental changes and real-time performance [118]. Fajrianti et al. (2023) employed SLAM with smartphone sensors for real-time location tracking after initializing the position via QR code, used Unity to obtain 3D environment data critical for Visual SLAM, and leveraged smartphone AR to display movement directions through the camera view [119]. However, these technologies have high requirements for mobile device performance, the tilt angle of the smartphone significantly affects navigation accuracy, and they also require a large number of QR codes to be deployed in the scene. In their other work, they propose an alternative method to reduce reliance on QR code recognition by leveraging object detection and optical character recognition (OCR) technologies to recognize information from naturally installed signs within the environment, thereby further enhancing the reliability of localization [120].

The vision-based indoor localization technology offers several advantages, including the elimination of base station deployment requirements, immunity to signal strength variations affecting positioning accuracy, and relatively cost-effective implementation. Additionally, the camera has the capability to capture a continuous stream of images, enabling real-time image processing and subsequent position calculation, ensuring timely updates of the target’s positional information. However, the research on the application of computer vision in indoor localization is still immature. Changes in the indoor environment, lighting conditions, and background interference may have an impact on the localization results, so appropriate preprocessing and correction are required. In addition, the image recognition algorithm and the localization solving algorithm used in localization still need to be further improved and supplemented to enhance the environmental generalization ability of the image recognition model and improve the universality of the model application.

#### 2.1.6. Acoustic Sensor-Based Indoor Localization Method

An acoustic sensor is a sensor used to detect and measure sound signals, which converts sound signals into electrical signals so that computers or other electronic devices can analyze and process the sound. An acoustic sensor in a smartphone is usually a microphone that enables the phone to perform functions such as audio signal reception, voiceprint recognition, acoustic fingerprint localization, and speech recognition. One of the main advantages of the indoor localization method based on acoustic sensors is that it can utilize the built-in microphone of the smartphone, eliminating the need for additional hardware devices while achieving highly accurate localization. This cost-effective solution holds great potential for indoor positioning.

According to the principle of localization, acoustic sensor-based indoor positioning Systems (AIPSs) can be classified into TOF, time of arrival (TOA), time difference of arrival (TDOA), DOA, and fingerprint-matching localization methods [121]. TOF and TOA-based systems require time synchronization between the anchor point and the smartphone, while TDOA-based systems do not. The TDOA-based localization system can achieve asynchronous localization, but it requires four base stations to be preset, and its localization accuracy and stability are weaker than those of the TOA architecture. A fingerprint-matching-based localization system requires the establishment of a fingerprint library, which is often time-consuming and costly to build and maintain. An overview of several acoustic signal-based localization methods is shown in Table 7.

Cricket, proposed by Priyantha et al. (2000), is the first indoor localization system that employs acoustics and utilizes TOF, which is actually a combination of acoustic and radio frequency (RF) signals with 12 cm accuracy, but it cannot be widely deployed due to its high noise level [129]. The system proposed by Chen et al. (2019) introduces a combined time division multiple access (TDMA) and frequency division multiple access (FDMA) transmission method for effectively detecting signal modes, but this method only selects two completely different sets of parameters as signal modes, making it challenging to further improve the update rate [124]. Zhang et al. (2019) applied fractional-order Fourier transforms to avoid the multipath effect, but it has high complexity [122]. Cao et al. (2020) proposed a detection algorithm based on the inter-correlation function, enabling the implementation of robust and real-time acoustic indoor localization systems [123]. However, the detection algorithm is compromised under low signal-to-noise ratio conditions. Bordoy et al. (2020) proposed two new methods for target localization without requiring manual measurements of the receiver position, knowledge of microphone position, or information about signal LOS. However, the system assumes that all the anchors are at a known altitude and can only achieve a two-dimensional localization [125]. Wang et al. (2021) proposed an acoustic fingerprinting-based indoor localization method for smartphones, which utilizes the differences in the frequency domain features of predetermined acoustic chirp signals at different locations. The average error is reported to be within 1 m in mixed LOS and NLOS indoor environments; however, the method exhibits limited accuracy under non-proximate target conditions [127]. Cheng et al. (2023) proposed a first-path detection algorithm to obtain reliable TDOA measurements, combining a maximum likelihood algorithm with TDOA measurements to obtain the location of the smartphone [126]. Li et al. (2024) used a two-step chirp signal detection algorithm with both coarse and fine searches, and the proposed coarse search demonstrates a remarkable success rate exceeding 99.9% in multipath and NLOS scenarios [130].

Indoor localization methods based on acoustic sensors have the advantages of high accuracy and compatibility; however, due to the shorter wavelength of the acoustic wave, it has a weaker penetration ability and is more susceptible to the multipath effect. In addition, the slower propagation speed of acoustic signals will be more sensitive to the Doppler effect, and the localization accuracy will be more affected. On the other hand, AIPS requires specific infrastructure to be installed in the environment, as well as more beacons, which can increase the deployment cost of the system.

#### 2.1.7. UWB-Based Indoor Localization Method

UWB is a pulsed communication technology; its high bandwidth and extremely short pulse waveform give it a better ability to penetrate targets compared to other technologies, and it has the advantages of high immunity to interference, high penetration capability, and high accuracy of ranging with multipath effect [131]. Currently, only some smartphones have built-in UWB chips, such as the iPhone 13 and iPhone 14 (Apple Inc., Cupertino, CA, USA) and the Samsung Galaxy S21 (Samsung Electronics Co., Suwon, Republic of Korea). UWB positioning usually requires beacons and an application that can be run on the smartphone to read the signals from these beacons. Therefore, there are some UWB-based indoor positioning methods in the field of indoor positioning, but there are fewer methods based on built-in UWB in smartphones. UWB communicates with pulses in very short time intervals, and its localization algorithms can be categorized into four types: TOA [132], TDOA [133], AOA [134], TOF [135], and RSS [136]. TOA and AOA are based on the radio transmission and reception time difference, enabling the accurate determination of both distance and direction angle. TDOA determines the position of a mobile node based on the time difference of signals received at different anchor points. Table 8 summarizes the characteristics of different UWB-based indoor localization methods.

The history of UWB dates back a hundred years to the invention of transoceanic wireless telegraphy by Popov and Marconi [141]. Tsang et al. (2021) proposed a localization method that integrates trilateration and fingerprinting to address the challenge of achieving cost-effective and accurate UWB-based positioning in complex indoor environments. [142]. Gong et al. (2021) used the time-delay matrix’s conjugate symmetry to extend the number of sample points and clusters to improve the estimation accuracy and the number of sources that can be identified, but it also increases the computational complexity [143]. Wang et al. (2024) designed a multilayered neural network that simultaneously addressed the errors in three coordinate components, along with three independent multi-layered neural networks dedicated to optimizing the errors in individual coordinate components. This innovative approach significantly mitigates the NLOS error of the UWB indoor positioning system. However, it should be noted that the complex network structure requires a long training time [139].

UWB technology is capable of achieving centimeter-level high-precision indoor positioning with excellent stability in the face of interference in complex indoor environments. However, its implementation necessitates costly infrastructure deployment and susceptibility to signal interference from liquids and metals. Additionally, incorrect configuration may result in interference from other systems operating within the ultra-wideband spectrum. Despite the excellent performance of UWB positioning, its widespread adoption as a ubiquitous indoor positioning solution remains challenging due to the associated high deployment costs.

#### 2.1.8. Other Indoor Location Methods

Besides the aforementioned indoor positioning methods, there are additional technologies applicable to smartphone-based indoor positioning, such as 5G, magnetic fields, LiDAR, visible light communication, and RFID. Although these techniques can be applied in the field of indoor localization in smartphones and have been thoroughly studied by scholars, they currently face various limitations that prevent their widespread application. Although 5G technology has been widely deployed and expanded its coverage in many cities, there are still areas with insufficient coverage. Moreover, the signal strength of 5G can be attenuated in complex indoor environments, leading to reduced positioning performance. Additionally, in many indoor scenarios, only one base station (called gNB) can be detected by the receiver, which limits the applicability of traditional geometric methods for indoor 5G positioning [144]. Magnetic field-based positioning uses natural or artificial magnetic signatures for localization. While it does not require extra infrastructure, its accuracy is low and variable due to distortions from metallic structures and electrical equipment. LiDAR technology is limited by the fewer number of smartphones that support it. Visible light communication’s reliance on LOS and susceptibility to ambient light conditions restrict its applicability in dynamic environments. RFID requires close proximity between the tag and reader for accurate tracking.

In summary, while these technologies present innovative approaches to indoor positioning, practical challenges related to infrastructure, cost, accuracy, and environmental dependencies currently limit their broad application. Continued research and technological advancements will be necessary to overcome these hurdles and fully realize the potential of these emerging methods in the realm of indoor positioning.

#### 2.1.9. Challenges

Currently, significant progress has been made in indoor positioning research based on smartphone sensors. Wi-Fi and BLE have attracted considerable attention from researchers due to their widespread application and relatively low deployment costs, making them extensively used within the indoor positioning industry. Inertial sensors, as built-in components of smartphones, represent another viable option with minimal cost. These methods can provide usable positioning accuracy at a lower cost, demonstrating broad research prospects and commercial promotion potential. At present, indoor positioning algorithms based on Wi-Fi fingerprinting, BLE RSS, and PDR are relatively mature, making them suitable for beginners in the field of smartphone-based indoor positioning. For those pursuing higher positioning accuracy, technologies such as UWB, BLE AOA, Wi-Fi RTT, AIPS, and visual SLAM offer greater precision but come with higher costs and more complex algorithms. However, with technological advancements and wider adoption, the application costs of these high-precision methods are expected to decrease, presenting significant potential for development. 

In terms of commercial applications, BLE technology, due to its low cost, ease of deployment, and maintenance, has significant commercial promotion potential. It is widely used in scenarios such as shopping malls, factories, hospitals, and museums. Vision-based AR positioning methods have also been applied in some shopping centers and airports. Meanwhile, sound signal-based positioning methods exhibit excellent performance in large venues and have been implemented in commercial applications at locations like airports, subway stations, and underground parking lots. UWB technology, with its high-precision positioning capabilities, is suitable for scenarios requiring accurate location tracking, such as asset finding and precise positioning tasks.

To summarize, the current single-sensor-based indoor positioning methods for smartphones still have many challenges, mainly including the following.

1. It is often difficult for a single sensor technology to provide continuous, high-precision localization services under all conditions. For example, Wi-Fi and BLE signals are highly affected by building structures, while visual or audio localization is susceptible to light conditions and noise levels.
2. Single sensors in localization have poor stability, such as the error accumulation problem of inertial sensors and the localization errors caused by the non-visual distance and multipath effect problems of BLE and Wi-Fi.
3. Deploying a high-density sensor network to achieve sufficient coverage and accuracy may increase the cost burden and implementation complexity, especially for indoor environments with large areas or complex structures.
4. Indoor environments change frequently, due to factors such as crowd movement and the addition of temporary obstacles, and it is often difficult for a single sensor solution to adapt to these changes in real-time, which affects the localization results.

To cope with these challenges, current research is gradually shifting to multi-source sensor fusion technology, which realizes complementary advantages by integrating data from multiple sensors, to achieve higher indoor positioning accuracy.

### 2.2. Fusion of Multi-Source Sensors for Indoor Localization of Smartphone

In the development process of modern indoor positioning technology, indoor positioning schemes based on a single sensor of a smartphone have significant limitations when applied. The inertial sensors built into smartphones exhibit excellent performance in short-term indoor positioning; however, the accumulation of positioning errors over time significantly impacts the accuracy of inertial sensors for both heading estimation and final position updates. RSSI ranging and fingerprinting methods based on wireless signals can achieve high positioning accuracy for a longer period of time. However, in complex indoor scenarios, their positioning accuracy lacks stability and is prone to fluctuations caused by multipath propagation NLOS, and other factors. Traditional single-sensor methods, while capable of providing a certain level of positional information, often face issues such as low accuracy and poor environmental adaptability. For instance, when using Wi-Fi positioning in large commercial centers, the signal can be affected by building structures and crowd density, leading to positional errors that may reach several meters. This clearly does not meet the requirements for high-precision applications. With the popularization of Internet of Things (IoT) devices and the increasing demand for more accurate positioning, multi-source sensor fusion technology is gradually becoming the mainstream trend in the field of indoor positioning. Integrating data from different types of sensors can significantly enhance the accuracy and reliability of positioning. Therefore, to improve the universality of indoor pedestrian localization application services, multi-source fusion methods are usually adopted to combine the advantages of existing localization sources and achieve more accurate indoor pedestrian localization by taking advantages of different localization sources. An overview of existing indoor localization methods by fusing multi-source sensors is shown in Table 9.

There are already several practical implementations of smartphone indoor positioning systems that utilize multi-source sensor fusion. Specifically, a successful case is the high-precision audio indoor positioning system deployed at Nanjing South Station in China, which integrates audio sensors and inertial sensors. By deploying audio base stations to emit sound signals of specific frequencies, the system calculates the time difference of signal propagation after the phone’s microphone receives the signals, achieving distance measurement. Combined with position updates from the phone’s inertial sensor to deduce user movement trajectories, it mitigates the accumulation of positioning errors caused by audio signal obstructions. Once fully deployed, this system will cover 560,000 square meters of indoor space at Nanjing South Station, including waiting halls, platforms, underground parking lots, etc. It uses BeiDou positioning outdoors, automatically switching to audio/radio frequency matrix signals indoors for seamless navigation, providing services such as meeting people, finding parked cars, and indoor navigation for travelers. Additionally, Apple’s AirTag location system combines UWB technology, BLE beacons, and crowdsourced data from the Find My network to achieve multi-level positioning from centimeter-level accuracy to wide-area coverage. AirTags continuously send BLE signals, and iPhones estimate approximate distances through BLE RSSI, with UWB assisting in precise direction finding. When users move with their iPhones, devices use VIO (visual inertial odometry) to calculate their own movement trajectory in real-time, combining multiple UWB ranging results to solve for the precise coordinates of the AirTag, achieving high-precision indoor positioning. Some large shopping malls also utilize Wi-Fi or BLE in conjunction with built-in inertial sensors in phones to assist customers in determining their locations and navigating to target stores, significantly enhancing the shopping experience. The smartphone indoor positioning system based on multi-source sensor fusion demonstrates its convenience in various scenarios such as train stations, hospitals, shopping centers, smart warehouses, museums, and underground parking lots. These applications highlight the significant advantages of multi-source fusion-based positioning systems in improving positioning accuracy, enhancing user experience, and optimizing operational processes.

Indoor localization methods fusing multi-source sensors have better robustness and accuracy and, thus, have higher feasibility in practical applications compared to a single localization source in complex indoor environments. Existing integration methods such as KF, EKF, UKF, and PF are typical methods for multi-source indoor localization fusion for indoor localization and navigation. Among them, KF is based on Bayesian filtering theory, which realizes accurate estimation of the system state by continuously fusing the a priori knowledge (system model) with the a posteriori measurements. However, the KF is primarily applicable to linear systems and assumes that both the process and measurement models are linear. When applied to non-linear systems, the KF may yield suboptimal or inaccurate results due to its inability to properly handle non-linearities. To address this limitation, the EKF expands on KF by approximating nonlinear system models and observation models as linear through a first-order Taylor expansion; however, EKF fails to handle systems with severe nonlinearity and non-Gaussian noise. On the other hand, the UKF can be applied to nonlinear systems but struggles with highly non-Gaussian noise. The true posterior distribution of the state of nonlinear and non-Gaussian systems can be approximated by employing particle filtering, which is based on Bayesian statistical theory and the sequential Monte Carlo framework. This approach is particularly well-suited for multimodal problems due to its ability to handle complex distributions, despite its computationally intensive nature.

#### 2.2.1. Fusion of Wi-Fi and Inertial Sensors for Indoor Localization

The indoor localization method of fusing Wi-Fi and inertial sensors aims to improve the accuracy and reliability of indoor localization by combining Wi-Fi signals and data from the IMU sensors built into smartphones. Wi-Fi localization has the advantages of wide coverage and ease of deployment, and it is usually capable of estimating the absolute position and can maintain high positioning accuracy for a long period of time. However, the estimated position is relatively discrete and is affected by NLOS, AP signal coverage, and multipath issues. Inertial sensor localization can only localize to a relative position with a known starting point, but the localized position is relatively continuous and does not depend on any particular signal coverage, and the error is small in a short period of time, but with the accumulation of time, the error will increase rapidly. Therefore, the fusion of Wi-Fi and inertial sensors is crucial for indoor localization as it leverages the continuous availability of inertial sensor localization results to rectify wireless signal errors, thereby compensating for their respective limitations. On the other hand, the absolute position obtained from the wireless signal is utilized to provide feedback to correct the PDR results. Depending on the Wi-Fi localization method, fusion localization methods can be classified into Wi-Fi RTT, RSSI ranging, and fingerprinting-based fusion localization methods. As shown in Table 10, there are many scholars who have proposed localization methods fusing Wi-Fi and inertial sensors in recent years.

The tightly coupled (TC) EKF method proposed by Sun et al. (2020) integrates Wi-Fi and PDR using the EKF algorithm for smartphone-based localization [145]. Liu et al. (2021) proposed a loosely coupled (LC) indoor localization method using EKF to fuse Wi-Fi RTT and PDR [146]. Choi (2021) proposed an EKF-based LC method for PDR and Wi-Fi range, and the fusion results were used for heading offset and step correction [147]. Zhou et al. (2024) proposed a multimodal sensor fusion algorithm for indoor localization with EKF that combines Wi-Fi fingerprints and IMU data to provide accurate and continuous pedestrian localization [148].

PF is also widely used in fusion systems of Wi-Fi and IMU data. Xu et al. (2019) proposed an enhanced particle filter with two different state-updating strategies for indoor localization by fusing the advantages of PDR and Wi-Fi FTM [149]. Chen et al. (2022) proposed a federated particle filter algorithm based on the principle of information sharing. The state transition equations included PDR and pedestrian heading, while the measurement equations incorporated step length and Wi-Fi matching results to improve pedestrian navigation accuracy [150]. Huang et al. (2023) integrated IMU and RSS fingerprinting through an improved particle swarm optimization-based algorithm [151]. The improved integrated localization algorithm developed by Lin et al. (2024) incorporates the PF with integrated PDR, Wi-Fi, and indoor navigation maps utilizing a location mapping model with BP neural networks to further reduce the error of the integrated localization method [152].

Wu et al. (2023) proposed a TC integration algorithm using a single Wi-Fi FTM AP to combine RSSI and PDR and used step error and heading error as error state vectors for PDR to enhance its accuracy. However, the coverage provided by a single Wi-Fi FTM AP is insufficient for application in a diverse range of scenarios [153]. Yang et al. (2023) utilized a fuzzy inference system to adaptively schedule Wi-Fi scans for Wi-Fi fingerprinting based on a rough metric that fuses the localization error and the residual energy of the smartphone, thus achieving a trade-off between localization accuracy and energy consumption [154]. Guo et al. (2023) proposed a tightly coupled fusion platform using RTT, RSS, and data-driven PDR to achieve a localization accuracy of 0.39 m [155]. Li et al. (2023) proposed a factor graph model with local attention (FGLA) and introduced the bounce gradient descent (BGD) algorithm to quickly optimize constrained FG in a non-vector space [156]. The fusion system utilizing PDR/Wi-Fi/barometer has verified that FGLA possesses stronger interference resistance when faced with sudden data anomalies. Xu et al. (2024) fused Wi-Fi and IMU data based on a deep learning approach using a co-teaching network to process noisy labels and a GAN-based domain adaptive module for IMU data labeling [157]. Sun et al. (2025) contribute a novel WiFi RTT/RSS/PDR/map fusion system with an enhanced map-aided particle filter, utilizing FTM-based fingerprinting and a semi-parametric error model to significantly reduce offline measurement efforts, improve localization accuracy, and optimize the particle filter’s performance [158].

Indoor positioning methods that integrate Wi-Fi and inertial sensors can utilize their respective advantages to achieve better positioning accuracy, but they still face some challenges. Wi-Fi signals are susceptible to reflections and attenuation in indoor environments. The establishment and maintenance of fingerprint databases are time-consuming and laborious. When pedestrians’ distance from the APs becomes large, the accuracy of Wi-Fi RSSI ranging decreases rapidly and is susceptible to interference in the external environment. The deployment of Wi-Fi APs requires more appropriate algorithms to maintain high positioning accuracy while reducing the number of deployments and, thus, minimizing the cost of the indoor positioning system.

#### 2.2.2. Fusion of BLE and Inertial Sensors for Indoor Localization

BLE and Wi-Fi are both wireless positioning methods. However, compared to Wi-Fi signals, indoor positioning methods based on BLE and inertial sensors offer advantages in terms of cost-effectiveness, power efficiency, and device compactness. Nonetheless, the coverage range of BLE is limited. The PDR method is characterized by short-term high accuracy, and it can detect and effectively eliminate BLE ranging errors due to the interference of the NLOS environment or similar frequency bands. Meanwhile, the location of normal signals obtained by BLE localization can be used to correct and suppress the error accumulation of the PDR method. The indoor localization methods fusing Bluetooth and inertial sensors can be classified into RSSI ranging-based, BLE AOA-based, and fingerprint-based fusion localization methods. Table 11 organizes some indoor positioning methods that fuse BLE and inertial sensors.

Dinh et al. (2020) proposed an improved RSSI trilateral localization method that can provide an accurate initial position of the PDR. In addition, a lightweight fingerprinting method was proposed to reduce the orbital drift of the PDR [159]. Chen et al. (2022) proposed a PF-based fusion algorithm that can effectively improve the accuracy of the indoor positioning system, and they optimized the inertial odometry network (IONet) framework by adding a coordinate frame matching module, which ultimately achieved an average position error of 1.76 m [160]. Ye et al. (2022) proposed a BLE AOA localization method based on signal fitting and propagator direct data acquisition angle estimation algorithms, eliminating the need for calculating phase difference between the antennas. This approach is applicable not only to linear antenna arrays but also to rectangular ones [161]. Jin et al. (2023) proposed a smartphone-based solution combining BLE and PDR in a PF framework, which achieves an average localization error of 1.34 m [162]. Guo et al. (2023) proposed pedestrian reachability and a floor map enhanced by smartphone virtual wireless devices for hybrid indoor real-time localization, achieving an average accuracy of 0.93 m [163]. The authors’ other work accomplished precise BLE ranging and position estimation through real-time monitoring of Bluetooth signals, incorporating the hybrid channel path loss model strategy, robust adaptive extended Kalman filtering, and pedestrian motion characteristics at the algorithmic level [164]. Liu et al. (2024) proposed an indoor localization method for smartphones that combines map localization anchors (MLA) with multi-sensor fusion fixing, and the proposed method with MLA-matched multi-source sensor localization module can achieve 1.01 m positioning accuracy in multi-floor scenarios with high robustness and usability [165]. Dyhdalovych et al. (2025) proposed a multi-carrier phase difference method for precise distance estimation based on BLE and, subsequently, integrated this with IMU data within a particle filter framework. This method is capable of reducing the impact of signal interference and multipath effects that commonly affect BLE positioning, thereby improving location accuracy [166].

The indoor positioning method of fusing Bluetooth and inertial sensors has become one of the most commonly used fused positioning methods, owing to its cost-effectiveness, ease of implementation, and the flexibility to deploy BLE beacons as required. The fusion of PDR data improves the positioning accuracy and stability of the BLE positioning system. However, similar to Wi-Fi signals, BLE signals can be affected by obstacle occlusion, radio interference, NLOS problems, and multipath effects, all of which contribute to an increase in positioning error. In contrast, with fingerprint-based methods, the localization accuracy depends greatly on the quality and size of the database.

#### 2.2.3. Fusion of Acoustic Signals and Inertial Sensors for Indoor Localization

Acoustic signals, due to their low synchronization rate, excellent performance, affordability, and high accuracy, have emerged as a prominent research area in fusion positioning technology. Especially in large-scale open indoor environments, the positioning system based on acoustic signals can achieve remarkable accuracy and are compatible with most intelligent devices. Compared with RF signals, acoustic signals have less terminal variability and excellent device compatibility. Indoor localization methods fusing acoustic sensors with inertial sensors obtain more accurate device location results by fusing the localization data from acoustic signals with the device position and attitude information collected by inertial sensors. In addition, the acoustic signals can provide a relative positional relationship between the sound source and the device, which is used to reduce the accumulated errors of the PDR method. These methods are described in Table 12.

Wang et al. (2019) proposed a joint localization algorithm fusing acoustic signals and inertial sensors to effectively improve the localization accuracy in the NLOS case [167]. Chen et al. (2021) proposed an indoor ranging solution for audible spectral acoustic signals less than 21 KHz, introducing the EKF to integrate inertial sensors and acoustic TDOA ranging data to achieve better positioning accuracy [168]. Xu et al. (2022) proposed a large-scale acoustic indoor positioning system integrating BLE nodes, acoustic modules, and MEMS sensors. The integrated acoustic propagation framework combining split-frequency multiple access, time-division multiple access, and space-division multiple access was used for acoustic signal enhancement, respectively, and the enhanced PF was used to further fuse the BLE and floor plan information [169]. Liu (2023) et al. obtained more accurate distance measurements in dynamic situations by transmitting dual chirp signals and achieved real-time localization accuracy at the decimeter level in some typical scenarios and a 4 Hz real-time positioning rate [170]. Guo et al. (2023) proposed low-cost anchor hardware, a two-step signal detection method, and a data-driven PDR, as well as an acoustic measurement compensation method and a measurement quality evaluation and control strategy, to improve the performance of position estimation [171]. Yan et al. (2023) proposed a fusion CHAN and the improved PDR indoor localization system that combines the CHAN algorithm and PDR algorithm to design an acoustic and PDR-based localization system, which improves PDR localization through step length estimation and heading direction estimation [172]. Wang et al. (2023) fused acoustic signals and IMU data using KF to take advantage of their respective strengths, and they used Bayesian parameter estimation to adjust the IMU parameters and to predict the motion trend, effectively addressing the challenge of prolonged unilateral data loss in conventional fusion localization [173]. Xu et al. (2025) proposed a novel 1D-CNN integrated with a channel attention mechanism (CAM) and long short-term memory (LSTM) for improved acoustic NLOS signal classification, enabling precise signal recognition through automated feature extraction and adaptation to diverse indoor environments [174].

Precise indoor positioning solutions fusing smartphone acoustic signals and inertial sensors still face the following challenges: unstable positioning accuracy on different smartphones in different environments; limited service coverage of the system due to the constraints of the effective detection distance and the transmission method; acoustic signals being susceptible to fading due to interference with obstacles in complex indoor environments; and an unusually high number of acoustic transmitters deployed, which leads to an increase in the system’s cost.

#### 2.2.4. Fusion of Vision and Inertial Sensors for Indoor Localization

The vast majority of smartphones nowadays are equipped with built-in cameras and inertial sensors, so the indoor positioning method that fuses cameras and inertial sensors has advantages due to its cost-effectiveness and elimination of the need for additional base stations. In addition, unlike RF localization, visual localization remains unaffected by complex RF signals in indoor environments, but visual localization is susceptible to the interference of lighting conditions and indoor obstacles, which affects the localization accuracy. More accurate indoor localization can be achieved by using the image information acquired by the camera for visual localization, combined with the attitude and motion information provided by inertial sensors. By fusing image and sensor data, the problems of the inability of cameras to acquire depth information and the tendency of inertial sensors to accumulate errors can be overcome. Table 13 summarizes the characteristics of different fusion methods.

Liu et al. (2017) proposed a constrained mobile localization method for indoor scenes in buildings, where the user can upload images of the scene taken by the smartphone to a server for identification and use a particle filtering algorithm to fuse the other sensor data of the phone for localization [175]. Neges et al. (2017) combined IMU and video images for indoor localization. IMU data were used to estimate the position and orientation of the mobile device and different semantic objects (e.g., exit signs and fire extinguishers) were extracted from the video to validate the obtained position [176]. Poulose et al. (2019) proposed a hybrid indoor localization system integrating a smartphone camera and IMU using a linear KF, where the camera pose reduces the IMU sensor error and the IMU localization result reduces the heading error of the camera localization [177]. Dong et al. (2022) proposed a smartphone-based visual-inertial odometry for indoor positioning-assisted pedestrian gait information, utilizing the PDR algorithm to establish two additional state constraints for the visual-inertial tracking system, and the visual-inertial odometry (VIO) system assisted the PDR algorithm for mode switching to improve the accuracy of the gait information by adaptive step size formulation [178]. Shu et al. (2022) used dynamic reliability curation to ensure the continuous robustness of the localization system based on the fusion of MEMS and image localization results [179]. Zheng et al. (2023) proposed an indoor visual positioning method with 3D coordinates using built-in smartphone sensors based on epipolar geometry, which transforms the localization problem into solving the spatial distance from a point to multiple lines, combining position information obtained from accelerometers and magnetometers with visual computation to obtain more accurate coordinates [180]. Bai et al. (2025) present a graph-based indoor 3D pedestrian location tracking method that solely utilizes the onboard inertial sensors of mobile devices for pedestrian state estimation in a SLAM mode [181].

Although fusing cameras and inertial sensors can improve the accuracy of indoor localization, the problem of localization errors still exists. The camera may be affected by factors such as light and occlusion, while the inertial sensor may suffer from the accumulation of cumulative errors. In addition, fused visual localization requires the performance of smartphones, while the computational resources and time required for camera and IMU data processing are substantial. Moreover, the generalizability of fused visual localization is low, and the localization method of visual recognition for scene awareness limits its extension to other indoor scenes.

#### 2.2.5. Other Integration Methods

Absolute positioning techniques, such as GNSS, Wi-Fi, BLE, UWB, and audio methods, are commonly employed to determine the precise position and orientation of a smartphone within a fixed reference system. The utilization of relative positioning techniques is currently prevalent in conjunction with inertial sensors, whereas visual positioning techniques can serve as both absolute and relative sources for indoor positioning [182]. Currently, multi-source fusion localization solutions using absolute localization techniques fused with inertial sensors are the more widely used, but some solutions use two absolute localization sources. Two wireless-based localization systems with similar physical characteristics can be integrated to act as mutual compensation. These efforts include Wi-Fi/BLE [183], RF/wireless sensor networks [184], and GNSS/5G [185,186]. Monica et al. (2019) proposed a hybrid localization method based on combining Wi-Fi and UWB, which provides sufficient accuracy without the cost of deploying the entire UWB infrastructure, providing a good compromise between accuracy and infrastructure cost [187]. Luo et al. (2019) proposed a hierarchical topological fingerprinting-based hybrid Wi-Fi and BLE indoor localization system, deploying BLE beacons in places where there is no clear Wi-Fi signal for localization, effectively improving the localization accuracy [188]. Peng et al. (2022) designed a fused localization system based on ultra-wideband and vision, using a Hungarian algorithm to match and a joint KF algorithm for fused localization [189]. Tang et al. (2024) used an adaptive multi-decision fusion mechanism to fuse Wi-Fi and image-based indoor localization methods, enabling the precise acquisition of the user’s location [190].

#### 2.2.6. Challenges

In the context of multi-source sensor fusion for indoor positioning using smartphones, the integration of Wi-Fi, BLE, and inertial sensors stands out due to the extensive use of existing infrastructure and lower deployment costs, offering promising commercial prospects. Moreover, fusion methods combining inertial sensors with visual or acoustic signals show significant potential in high-precision applications. Currently, filter-based fusion approaches have relatively mature research algorithms, making them ideal for beginners to study, research, and further refine to enhance positioning accuracy. Concurrently, graph optimization-based methods and learning-based approaches have garnered considerable attention in recent years. These methods each exhibit unique advantages and hold substantial development potential within the realm of multi-sensor fusion positioning.

Although many scholars have proposed indoor localization solutions for fusing multi-source sensors and achieved good localization performance, it is still difficult recommend a unified approach in terms of accuracy, cost, positioning time, and system complexity when applied to actual applications.

1. Existing multi-source fusion positioning solutions are usually optimized for a certain type of indoor scene or a specific user to achieve good positioning performance. However, the diversity of indoor environments introduces unique layouts, structures, and walking modes that can potentially impact the accuracy of indoor localization.
2. Given the widespread use of Wi-Fi and BLE, a growing number of indoor positioning systems incorporate wireless positioning technologies alongside IMU integrated into smartphones. This approach offers extensive coverage and system capacity; however, its accuracy is susceptible to multipath interference and NLOS conditions.
3. To improve indoor positioning accuracy, the existing methods are often realized by increasing the number of sensors, amount of data, and algorithm complexity, but this will also lead to higher implementation costs and operational and maintenance expenses for the system.
4. Some fusion positioning systems, which lack inertial sensors, can utilize the complementary physical characteristics of wireless technologies to compensate for each other and serve as a class of indoor positioning solutions. However, these systems face challenges in acquiring velocity and attitude information, similar to inertial sensors, and may be influenced by low sampling rates.

## 3. Conclusions and Discussion

In this paper, we first briefly summarize the working mechanisms of different indoor positioning techniques and review the research progress of indoor positioning techniques for smartphones as well as multi-source sensor fusion positioning systems. Our aim is to provide practitioners and researchers with a comprehensive understanding of the current status and progress of the research in this field, while elucidating the overall development direction of smartphone-based multi-sensor fusion positioning techniques. 

Currently, fused positioning methods have made significant advancements in both research and application within the domain of smartphone indoor positioning. However, several challenges persist, offering clear opportunities for further enhancement. Many remaining challenges can be addressed by focusing research in the following directions:

1. Building maps can be constructed to constrain the indoor positioning of cell phones, constraining and matching the indoor positioning results by the a priori information of the building maps as well as the geometrical semantic and positional information contained in the building maps, so as to correct the coordinate information of the indoor positioning. In addition, accurate identification of the entrance location and floor identification of the building helps to construct a seamless indoor and outdoor localization system.
2. The deployment of additional facilities required in an indoor positioning system, such as wireless access points and BLE beacons, is also an important part of the system. The deployment location of these devices affects both the system implementation cost and positioning accuracy. By improving the wireless beacon location deployment algorithm to determine the optimal location for each beacon, the coverage of the wireless signals can be fully utilized. This reduces the number of wireless beacons needed, lowers the system cost, and reduces the measurement noise of the signals.
3. Multipath and NLOS issues seriously affect the accuracy of wireless localization. Future work may focus on detecting and mitigating these problems in integrated systems. Signal-processing algorithms can be implemented to identify and filter out multipath components, thereby improving the quality of received signals. Machine learning models can be trained using historical datasets to recognize patterns indicative of multipath and NLOS conditions. Furthermore, multi-source sensors can aid in the detection of multipath and NLOS through environmental awareness or by cross-verifying measurement results.
4. PDR systems based on artificial intelligence represent a forward-looking research field. The grip position of smartphones can introduce errors in the heading estimation of PDR systems. To mitigate this, data from the smartphone’s built-in accelerometer, gyroscope, and magnetometer can be used to train machine learning models to detect user behaviors such as holding the phone in hand, in a pocket, or in a bag, thereby adjusting the heading estimation accordingly. Context-aware algorithms can also dynamically correct headings based on real-time behavior and the environment. Furthermore, by incorporating a big data artificial intelligence mechanism for the adaptive adjustment of step length estimates, individual differences like gender, body type, and height can be considered, thus improving the accuracy of indoor positioning.
5. Some indoor localization methods are only oriented to a certain type of indoor scene, but indoor environments are complex and varied, including rooms, corridors, halls, staircases, elevators, large arenas, warehouses, underground parking lots, and so on. The behavior of pedestrians is different in different indoor scenes, and the operation characteristics of the built-in sensors of smartphones are also different. Accurately identifying and sensing complex indoor scenes and optimizing their positioning weights for each sensor module in different scenes can help achieve high-precision indoor positioning in complex indoor environments.
6. To achieve optimal position estimation, the integration of multiple localization technologies is crucial. Beyond traditional filtering algorithms for fusing positioning information from multiple sensors, methods based on graph optimization and deep learning have also been extensively researched. Graph optimization techniques can more accurately handle nonlinear and multimodal issues, making them particularly suitable for long-term tracking or complex environmental localization tasks. Deep learning approaches offer unique advantages in addressing multipath effects, NLOS errors, and dynamic environmental changes. Future research should focus on developing adaptive fusion techniques that not only automatically adjust parameters based on real-time environmental changes but also efficiently manage resource-constrained conditions, especially for mobile devices like smartphones.

In conclusion, the use of a single indoor positioning technique alone is no longer sufficient to meet the stringent requirements for positioning accuracy, system stability, coverage, cost, power consumption, and deployment complexity in smartphone-based indoor positioning systems. To address these challenges, researchers have begun combining two or more indoor positioning techniques. By leveraging the unique characteristics of different positioning sources, these hybrid approaches aim to complement each other and reduce positioning errors. However, even with dual-source fusion positioning methods, achieving satisfactory levels of stability, reliability, and cost-effectiveness remains challenging. Therefore, researchers are now exploring more advanced data processing and fusion algorithms to enhance multi-source sensor fusion methods. These advancements include improving the performance of filtering algorithms to increase positioning stability, introducing deep learning methods for sensor fusion, utilizing multi-sensors to detect multi-paths and NLOS conditions, and enhancing the positioning algorithms of different sensors or exploring new sensors for positioning, such as the high-reliability and low-latency Spark technology. Additionally, some scholars have fused three or more localization sources to achieve better localization accuracy and stability, such as fusing Wi-Fi/magnetic field/PDR and acoustic ranging/Wi-Fi/INS. However, more localization sources increase the system complexity and implementation cost while improving the localization accuracy. Despite the inherent limitations of current fusion localization methods, these techniques show significant potential for further development. Given that smartphones are the most commonly used mobile devices, high-precision localization technologies based on smartphones and their built-in sensors will remain a prominent research focus in the foreseeable future.

## Figures and Tables

**Table 1:** Comparison of related surveys.

Author	Time	Survey Contents
Morar et al. [30]	2020	An overview of the field of computer vision-based indoor localization
Kunhoth et al. [31]	2020	Different computer vision-based indoor navigation and localization systems are reviewed
Liu et al. [32]	2020	Distance-based acoustic indoor localization is divided into absolute and relative distance localization
Guo et al. [33]	2020	Fusion-based indoor localization techniques and systems with three fusion features: source, algorithm, and weight space
Liu et al. [34]	2020	Existing RF-based indoor localization systems are reviewed
Ashraf et al. [35]	2020	Reviewed methods for estimating a user’s indoor location using data from smartphone sensors
Simões et al. [36]	2020	Indoor navigation and positioning system for the Blind
Pascacio et al. [37]	2021	A systematic review of cooperative indoor localization systems
Obeidat et al. [38]	2021	Indoor positioning technologies and wireless technologies are reviewed
Hou and Bergmann [39]	2021	A systematic review and quality assessment of research on PDR and wearable sensors
Ouyang and Abed-Meraim [40]	2022	Reviewed magnetic fingerprint localization techniques
Aparicio et al. [41]	2022	Summarizes the main characteristics of acoustic positioning systems in terms of accuracy, coverage area, and update rate
Wang et al. [42]	2022	A systematic review of smartphone-based inertial localization and navigation methods is presented
Chen and Pan [43]	2024	Reviewed work related to inertial localization based on deep learning
Naser et al. [44]	2023	A systematic compendium and analysis of smartphone-based indoor localization methods
Zhuang et al. [45]	2023	An overview of combined multi-sensor navigation/positioning systems is presented

**Table 2:** Comparison of indoor localization methods for single positioning systems.

Systems	Equipment	Positioning Methods	Accuracy	Advantages	Limitations	Costs
Wi-Fi	Wi-Fi sensors, Wi-Fi AP	RSSI/Fingerprinting/Wi-Fi RTT	3–10 m	No additional infrastructure is required, Wide coverage	Cumbersome fingerprint database creation, Susceptible to signal interference and blockage, Fewer devices supporting Wi-Fi RTT protocols	Medium
BLE	Bluetooth sensors, Bluetooth beacons	RSSI/Fingerprinting/Proximity/AOA	1–5 m	low power consumption, Easy deployment, Small device size	Smaller range, Susceptible to signal interference, Poorer signal stability	Low
Inertial navigation	Inertial sensors (accelerometer, gyroscope, magnetometer)	INS/PDR/Motion constraints	Decreases with increasing positioning time	Sensors built into smartphones, No signal interference	Problems with error accumulation	Low
Barometer	Barometer sensors	Barometric floor positioning	Meter scale	No additional equipment to deploy	Vulnerability to external factors	Low
Vision	Camera	Feature detection/Visual marker/SLAM	Decimeter scale to Meter scale	No need for base station deployment, Not affected by signal strength	Susceptible to light conditions and background interference	Low
Acoustic	Acoustic sensors, Signal transmitter	TOF/TOA/TDOA/DOA	Depends on the distribution density of the infrastructure	Good compatibility, High scalability, High accuracy potential	More sensitive to the Doppler effect, Small beacon coverage area, Cumbersome fingerprint database creation	High
UWB	UWB base station, UWB receiver	RSSI/TOA/TDOA/AOA	Centimeter scale	Low power consumption, Insensitive to multipath effects	Liquids and metallic materials can block signals, Relatively high cost of hardware devices	Medium
5G	5G base station, 5G antenna	RSSI/TOA/TDOA/AOA/CSI	Meter scale	High-ranging accuracy and reliability	Signal susceptibility to interference	Low
Magnetic	Magnetometer	Fingerprinting	Meter scale	No need to deploy additional equipment	Poor generalizability, Susceptible to indoor magnetic interference	Low

**Table 3:** Wi-Fi-based indoor localization methods.

Method	Time	Author	Research Focus
RSSI	2019	Amri et al. [49]	A fuzzy localization algorithm calculates the distance between the anchor point and the sensor node using RSSI measurements.
	2023	Tao et al. [50]	Extreme value-based access point (AP) selection and localization algorithm
	2023	Vishwakarma et al. [51]	Classification of specific locations into specific regions based on graph neural network (GNN) and collected RSSI values
Fingerprinting	2017	Wang et al. [52]	Deep learning based fingerprinting (DeepFi), a deep learning-based indoor fingerprint localization method
	2018	Xu et al. [28]	Utilize indoor environment constraints in the form of a grid-based indoor model to improve the localization of a Wi-Fi-based system.
	2022	Lan et al. [53]	Super-resolution-based fingerprint enhancement framework for fingerprint enhancement as well as super-resolution fusion
	2023	Wang et al. [54]	Three-dimensional dynamic localization model based on temporal fingerprinting
	2023	Hosseini et al. [55]	A method for generating virtual fingerprints of building interiors by predicting Wi-Fi RSS values using integration of building information modeling (BIM) and signal propagation
	2024	Kargar-Barzi et al. [48]	Lightweight indoor Wi-Fi fingerprint localization method based on convolutional neural network (CNN) and convolutional self-encoder
	2024	Pan et al. [56]	Indoor Wi-Fi localization fingerprint database construction method based on crow search algorithm optimized density-based spatial clustering of applications with noise and recurrent conditional variational autoencoder-generative adversarial network
Wi-Fi RTT	2020	Huang et al. [57]	Learning nonlinear mapping relationship between indoor location and Wi-Fi round-trip time (RTT) ranging information using deep convolutional neural network
	2020	Yu et al. [58]	A Wi-Fi RTT-based data acquisition and processing framework for reducing multipath and non-line of sight (NLOS) errors
	2024	Guo et al. [59]	Clock drift error reduction based on clock drift theory modeling localization system framework, state monitoring algorithms, and partial differential equation constraint models
	2024	Cao et al. [60]	Wi-Fi RTT localization method based on line of sight (LOS) compensation and trusted NLOS identification

**Table 4:** Bluetooth-based indoor localization methods.

Method	Author	Time	Research Focus
RSSI	2021	You et al. [65]	RSSI-based multipoint localization algorithm
	2023	Gentner et al. [66]	Position is calculated on the server using particle filtering and returned to the mobile device
	2023	Assayag et al. [67]	Adaptive path loss model
	2024	Wu et al. [68]	Using KF to attenuate the effect of random perturbations
Fingerprinting	2023	Safwat et al. [69]	K-nearest neighbor (KNN) and weighted k-nearest neighbor (WKNN) based fingerprint localization methods
	2023	Shin et al. [70]	Fingerprint mapping method based on RSS sequence matching
	2024	Junoh et al. [62]	Generative adversarial network (GAN)-based semi-crowdsourced fingerprint map construction method for labor reduction
AOA	2023	Xiao et al. [71]	Improving AOA estimation accuracy by estimating phase noise using the extended Kalman filter
	2024	Wan et al. [72]	Improved signal subtraction subspace algorithm to reduce interference from coherent signals and errors caused by movement between people in the room
Proximity	2015	Zhao et al. [63]	BLE proximity detection based on particle filtering
	2020	Spachos et al. [73]	BLE proximity detection and RSSI-based localization

**Table 5:** PDR-based indoor localization methods.

Author	Time	Research Focus
Klein et al. [97]	2018	Machine learning classification algorithm to recognize smartphone modes
Guo et al. [98]	2019	Adaptive walking speed estimation for smartphone based on attitude sensing
Zheng et al. [99]	2020	Heading estimation algorithm for pocket and swing modes
Yao et al. [100]	2020	Step detection and step length estimation algorithms for recognizing different walking modes
Zhang et al. [101]	2021	A low-cost indoor navigation framework combining inertial sensors and indoor map information
Zhao et al. [102]	2023	Denoising MEMS data using bias drift model and KF
Wu et al. [103]	2024	PDR algorithm for multi-sensor fusion based on particle filter (PF)-UKF
Liu et al. [104]	2024	Extended Kalman filter (EKF)-based integration method for pedestrian motion constraints, smartphone sensors, and step detection methods
Chen et al. [105]	2024	3D localization method based on terrain feature matching

**Table 6:** Vision-based indoor localization methods.

Method	Time	Author	Research Focus
Image Match	2020	Li et al. [111]	Accurate single-image-based indoor visual localization method
	2020	Kubícková et al. [112]	Scale-invariant feature transform (SIFT) algorithm for feature detection and matching to find coordinates of image database using perspective-n-point (PnP) method
	2021	Li et al. [113]	Deep belief network-based scene classification and PnP algorithm to solve camera position
Object Detection	2018	Xiao et al. [114]	Deep learning-based localization method for large indoor scenes
	2021	Jung et al. [115]	Deep learning-based matching of object position and pose
	2024	Chen et al. [116]	Landmark matching method to match the landmark within an up-view image with a landmark in the pre-labeled landmark sequence
Visual Marker	2020	Tanaka et al. [117]	An ultra-high precision visual marker with pose error less than 0.1°
SLAM	2021	Xu et al. [118]	A visual simultaneous localization and mapping (SLAM)-based infrastructure-free indoor navigation system
	2023	Fajrianti et al. [119]	Unity for 3D environment modeling, visual SLAM with a smartphone’s gyroscope and camera for real-time tracking
	2024	Fajrianti et al. [120]	By using object detection technologies to identify information from naturally installed signs on-site

**Table 7:** Acoustic signal-based indoor localization methods.

Method	Time	Author	Research Focus
TOA	2019	Zhang et al. [122]	TOA estimation method for extracting first path signal based on the iterative cleaning process
	2020	Liu et al. [121]	TOA estimation method for smartphone based on built-in microphone sensor
	2020	Cao et al. [123]	A novel TOA detection algorithm for acoustic signals consisting of coarse search and fine search
TDOA	2019	Chen et al. [124]	Doppler shift-based TDOA correction method
	2020	Bordoy et al. [125]	TDOA measurement method without manual measurement of receiver position
	2023	Cheng et al. [126]	Maximum likelihood algorithms combined with TDOA measures
Fingerprinting	2021	Wang et al. [127]	Detection of the first path based on time-division multiplexing, utilizing power spectral density (PSD) of the frequency domain signal as a fingerprinting feature
	2024	Xu et al. [128]	Constructing an audio-chirp-attention network model fusing edge detection maps with normalized energy density maps and correlating fingerprint datasets with corresponding spatial locations

**Table 8:** UWB-based indoor localization methods.

Method	Time	Author	Research Focus
TDOA	2019	Pan et al. [133]	Improved TDOA and KF to compute the position of target nodes
	2021	Bottigliero et al. [137]	No need for time synchronization between sensors, using a unidirectional communication method to reduce the cost and complexity of tags
AOA	2021	Monfared et al. [134]	Iterative AOA localization algorithm for multilevel anchor selection under NLOS conditions
	2024	Zhong et al. [138]	AOA-based position tracking system and data processing algorithms to minimize system static error
DOA	2021	Gong et al. [139]	Frequency doubling and cluster counting algorithm for joint estimation of TOA and DOA
TOF	2020	Li et al. [135]	A neural network approach has been adopted to enhance the system’s performance in NLOS scenarios.
RSS	2022	Chong et al. [140]	Integration of UWB RSS into Wi-Fi RSS fingerprinting-based indoor localization system

**Table 9:** Overview of indoor localization method for fusion of multi-source sensors.

System	References	Cost	Strengths/Weaknesses
Wi-Fi/PDR	[145,146,147,148,149,150,151,152,153,154,155,156,157,158]	Medium	Wi-Fi positioning results provide accurate initial positioning, fusing PDR for position update and reducing the cumulative error of PDR. However, fewer devices support the Wi-Fi RTT protocol and are susceptible to signal interference and blockage.
BLE/PDR	[159,160,161,162,163,164,165,166]	Low	Bluetooth positioning results provide accurate initial positioning and fusion of PDR for position updating to reduce the cumulative error of PDR. The lower cost and power consumption of BLE and PDR are suitable as a pervasive indoor positioning method, but the fusion method has less coverage and is susceptible to signal interference.
Acoustic/PDR	[167,168,169,170,171,172,173,174]	High	Acoustic signals can provide the relative positional relationship between the sound source and the device, which is used to reduce the accumulated error of the PDR method. However, acoustic signals are susceptible to the Doppler effect and are easily blocked and absorbed by obstacles in complex indoor scenes, and high accuracy can be achieved by deploying sufficient devices in large, more open scenes.
Vision/PDR	[175,176,177,178,179,180,181]	Low	Visual localization using the image information acquired by the camera, combined with the attitude and motion information provided by the PDR, can reduce the cumulative error of the PDR. Visual localization is not subject to signal interference, but is susceptible to lighting conditions and background interference, and requires high smartphone performance.

**Table 10:** Fusion of Wi-Fi and inertial sensors for indoor localization.

Author	Time	Research Focus
Xu et al. [149]	2019	Enhanced PF with two different state update strategies and fast reinitialization
Sun et al. [145]	2020	Least-squares (LS)-based real-time ranging error compensation model and weighted least-squares (WLS)-based adaptive Wi-Fi FTM localization algorithm
Liu et al. [146]	2021	Adaptive filtering system consisting of multiple EKFs and outlier detection methodology
Choi et al. [147]	2021	Calibration-free localization using Wi-Fi ranging and PDR
Guo et al. [14]	2022	A tightly coupled method based on Wi-Fi RTT, RSSI, and MEMS-IMU
Chen et al. [150]	2022	Federated particle filter (FPF) fusion of PDR and Wi-Fi based on information sharing principle
Huang et al. [151]	2023	Improved particle swarm optimization-based algorithm for integrating inertial sensors and RSS fingerprinting
Wu et al. [153]	2023	Using only one Wi-Fi FTM AP and estimating position with the smartphone’s built-in inertial sensor
Yang et al. [154]	2023	Fuzzy logic-based fusion localization method adaptively schedules energy-consuming Wi-Fi scans
Guo et al. [155]	2023	Tightly coupled fusion platform for Wi-Fi RTT, RSS, and data-driven PDR based on factor graph optimization
Li et al. [156]	2023	The factor graph (FG) model with local attention can constrain factor nodes within the graph to quickly correct local outliers
Lin et al. [152]	2024	Enabling PF integration of PDR, Wi-Fi, and indoor maps
Zhou et al. [148]	2024	EKF-based multimodal sensor fusion algorithm for indoor localization
Xu et al. [157]	2024	Enhancing Wi-Fi fingerprint localization with a co-teaching approach using crowdsourced sequential RSS and IMU data
Sun et al. [158]	2025	Utilizing an improved map-aided PF to fuse WiFi RTT, RSS, PDR, and map information

**Table 11:** Fusion of BLE and inertial sensors for indoor localization.

Author	Time	Research Focus
Dinh et al. [159]	2020	Estimating approximate distance methods to estimate initial position and lightweight fingerprinting methods
Chen et al. [160]	2022	Data-driven integration of BLE-based inertial navigation using PF
Ye et al. [161]	2022	Angle estimation algorithm based on signal fitting and propagator direct data acquisition
Jin et al. [162]	2023	PF-based indoor localization framework for BLE and PDR
Guo et al. [163]	2023	Hybrid indoor localization approach with pedestrian reachability and floor map constraints based on virtual wireless devices
Guo et al. [164]	2023	Robust adaptive EKF-based multi-level constraint fusion localization framework
Liu et al. [165]	2024	A smartphone indoor localization method that fuses map positioning anchors with multi-sensor fusion
Dyhdalovych et al. [166]	2025	Utilizing a multi-carrier phase difference method for precise distance estimation based on BLE

**Table 12:** Fusion of acoustic signals and inertial sensors for indoor localization.

Author	Time	Research Focus
Wang et al. [167]	2019	Positioning system combining acoustic signals and IMUs to correct NLOS errors
Chen et al. [168]	2021	Introduction of EKF to integrate IMU and acoustic TDOA ranging data
Xu et al. [169]	2022	Hybrid acoustic signal transmission architecture based on frequency division multiple access, time division multiple access, and space division multiple access
Liu et al. [170]	2023	Low-cost, large-scale indoor positioning system based on audio dual chirp signals
Guo et al. [171]	2023	Acoustic measurement compensation method and measurement quality assessment and control strategy
Yan et al. [172]	2023	Fusion of CHAN and improved PDR indoor localization system
Wang et al. [173]	2023	Fusion of acoustic signals and IMU data using KF
Xu et al. [174]	2025	Acoustic NLOS signal recognition based on 1-D CNN with channel attention mechanism

**Table 13:** Fusion of vision and inertial sensors for indoor localization.

Author	Time	Research Focus
Liu et al. [175]	2017	A multi-sensor fusion approach for camera, Wi-Fi, and inertial sensors on smartphones
Neges et al. [176]	2017	Indoor navigation system based on IMU and real-time visual video streaming AR technology
Poulose et al. [177]	2019	Indoor positioning method using smartphone IMU, Wi-Fi RSSI, and camera
Dong et al. [178]	2022	Visual inertial mileage assisted by pedestrian step information
Shu et al. [179]	2022	Efficient image-based indoor positioning using MEMS
Zheng et al. [180]	2023	An indoor visual positioning method with 3D coordinates using built-in smartphone sensors based on epipolar geometry
Bai et al. [181]	2025	Mobile devices use their onboard inertial sensors alone for pedestrian state estimation in SLAM mode

## Data Availability

Not applicable.
